# Adult Prey Neutralizes Predator Nonconsumptive Limitation of Prey Recruitment

**DOI:** 10.1371/journal.pone.0154572

**Published:** 2016-04-28

**Authors:** Julius A. Ellrich, Ricardo A. Scrosati, Katharina Romoth, Markus Molis

**Affiliations:** 1 Department of Biology, St. Francis Xavier University, Antigonish, Nova Scotia, Canada; 2 Department of Chemistry and Biology of the Marine Environment, Carl von Ossietzky Universität, Oldenburg, Niedersachsen, Germany; 3 Section Functional Ecology, Alfred-Wegener-Institut, Helmholtz-Zentrum für Polar- und Meeresforschung, Bremerhaven, Bremen, Germany; The University of Sydney, AUSTRALIA

## Abstract

Recent studies have shown that predator chemical cues can limit prey demographic rates such as recruitment. For instance, barnacle pelagic larvae reduce settlement where predatory dogwhelk cues are detected, thereby limiting benthic recruitment. However, adult barnacles attract conspecific larvae through chemical and visual cues, aiding larvae to find suitable habitat for development. Thus, we tested the hypothesis that the presence of adult barnacles (*Semibalanus balanoides*) can neutralize dogwhelk (*Nucella lapillus*) nonconsumptive effects on barnacle recruitment. We did a field experiment in Atlantic Canada during the 2012 and 2013 barnacle recruitment seasons (May–June). We manipulated the presence of dogwhelks (without allowing them to physically contact barnacles) and adult barnacles in cages established in rocky intertidal habitats. At the end of both recruitment seasons, we measured barnacle recruit density on tiles kept inside the cages. Without adult barnacles, the nearby presence of dogwhelks limited barnacle recruitment by 51%. However, the presence of adult barnacles increased barnacle recruitment by 44% and neutralized dogwhelk nonconsumptive effects on barnacle recruitment, as recruit density was unaffected by dogwhelk presence. For species from several invertebrate phyla, benthic adult organisms attract conspecific pelagic larvae. Thus, adult prey might commonly constitute a key factor preventing negative predator nonconsumptive effects on prey recruitment.

## Introduction

Predators control prey populations by killing prey, but they also have nonconsumptive effects (NCEs) on prey [1]. NCEs are often triggered by chemical or visual predator cues that are detected by prey [2,3]. Upon cue detection, immediate prey responses often include moving away or decreasing feeding activities to minimize predation risk [4–9]. Such responses occur in aquatic and terrestrial predator—prey systems [10,11]. As predator cues may reach many prey individuals at the same time, NCEs may have larger consequences for prey populations than consumptive effects, as indicated by theoretical [12,13] and empirical [14–17] studies. For this reason, currently an important aim in ecology is to identify what factors affect the intensity of predator NCEs on prey [18].

Studies on invertebrate predator—prey systems have found that predator cues can limit prey larval settlement [3,19–21] and subsequent recruitment [22], as a number of settling larvae move away when predator cues are detected to reduce future predation risk [23–25]. However, studies using species from several groups, including molluscs, polychaetes, echinoderms, arthropods, and tunicates, have found that benthic adult organisms chemically attract conspecific pelagic larvae that are seeking habitat for settlement [26–31]. Such a behavior is thought to enhance the long-term persistence of populations, as the attraction exerted by adults guides larvae to locate adequate conditions for development [30,32,33]. Therefore, for species in which adults attract conspecific larvae, the presence of adult organisms might reduce, or even eliminate, predator NCEs on the recruitment of such prey species. This study experimentally investigates this notion using marine predators (dogwhelks) and prey (barnacles) as a model system.

Barnacles are sessile organisms with pelagic larvae and are common in intertidal habitats worldwide [34]. Dogwhelks are benthic predatory snails that frequently feed on intertidal barnacles [35,36]. Barnacle larvae often react negatively to chemical cues released by dogwhelks (e.g., pedal mucus [19]). Recent field experiments have shown that, in the absence of adult barnacles, waterborne chemical cues from dogwhelks can limit barnacle larval settlement [21] and, ultimately, barnacle recruitment [22]. However, adult barnacles attract conspecific larvae that are seeking settlement [37–40] through chemical [41–45] and visual [46] cues, in that way enhancing barnacle recruitment [47–49]. Therefore, we conducted a factorial field experiment that simultaneously manipulated the presence of dogwhelks and adult barnacles to test the hypothesis that adult barnacles can neutralize the negative NCEs that dogwhelks have on barnacle recruitment. To examine the generality of this prediction, we replicated the experiment in two years.

## Material and Methods

### Study System

For barnacles, settlement is the permanent contact with the substrate established by pelagic cyprid larvae [50], while recruitment is the appearance of new benthic individuals that have metamorphosed after larval settlement and have reached a size that allows them to be counted [51]. We did the experiment in rocky intertidal habitats on Deming Island (45° 12' 45" N, 61° 10' 26" W), near Whitehead, on the Atlantic coast of Nova Scotia, Canada. The experiment spanned two barnacle recruitment seasons (2012 and 2013), that is, the period during which recruits appeared on the shore. Daily maximum water velocity (an indication of wave exposure) determined with dynamometers (see design in [52]) was 5.0 ± 0.7 m s^-1^ (mean ± SE, range = 3.5–6.6 m s^-1^; n = 5) in the 2012 recruitment season and 4.2 ± 0.1 m s^-1^ (range = 3.0–6.9 m s^-1^; n = 94) in the 2013 recruitment season. Thus, the studied habitats were subjected to a moderate wave action, since habitats directly facing the open ocean in Nova Scotia experience water velocities up to 12 m s^-1^ [53]. Intertidal temperature measured every 30 minutes throughout consecutive high and low tides with submersible loggers (HOBO Pendant Logger, Onset Computer Corp., Pocasset, MA, USA) was 9.2 ± 0.2°C (mean ± SE; n = 6 loggers) during the 2013 recruitment season (no data are available for 2012), with temperatures not exceeding 20°C during low tides. Coastal seawater salinity was 30 ppt in both years [54]. The abundance of coastal phytoplankton (food for barnacle nauplius larvae and recruits [34, 55]) measured as chlorophyll-*a* concentration was 1.50 ± 0.49 mg m^-3^ (mean ± SE; n = 3) during the 2012 recruitment season and 3.22 ± 0.02 mg m^-3^ (n = 2) during the 2013 recruitment season (MODIS-Aqua satellite data [56]).

On this coast, *Semibalanus balanoides* (L. 1767) is the only intertidal barnacle species [57]. It is a cross-fertilizing hermaphrodite [34,58] that broods once per year [59,60]. In Atlantic Canada, *S*. *balanoides* mates in early autumn, breeds in winter, and releases pelagic larvae in spring [59,61]. Larvae develop over 5–6 weeks in the water column [59]. In northern Nova Scotia, barnacle recruits appear in intertidal habitats in May and June [49]. The dogwhelk *Nucella lapillus* (L. 1758) is the main predator of *S*. *balanoides* on this coast. Movement and feeding in *N*. *lapillus* start at 3–5°C of water temperature and increase up to 20°C [62]. On the Atlantic coast of Nova Scotia, *N*. *lapillus* becomes active in April [35,63], when it can be found preying on barnacles. Under the environmental conditions described above, *N*. *lapillus* cues limit *S*. *balanoides* recruitment [22] by limiting larval settlement [21]. *Nucella lapillus* releases pedal mucus during locomotion [64].

### Field Experiment

To test our hypothesis, we did a manipulative field experiment. Both in 2012 and 2013, we used "dogwhelks" and "adult barnacles" as crossed factors, each with two levels (presence and absence), arranged following a randomized complete block design with each of the four treatments replicated twice within each block. We established six blocks each year on relatively horizontal intertidal areas, totalling 24 experimental units (12 per year) for each of the four treatments involving the "dogwhelks" and "adult barnacles" factors. We used different blocks in each year. The vertical intertidal range is 1.8 m on this coast and the blocks were established at an elevation range of 0.7–1.4 m above chart datum (lowest normal tide in Canada). Block size was 15.3 ± 3.0 m^2^ (mean ± SE; n = 12 blocks), with experimental units being at least 0.5 m apart within blocks.

**Fig 1:**
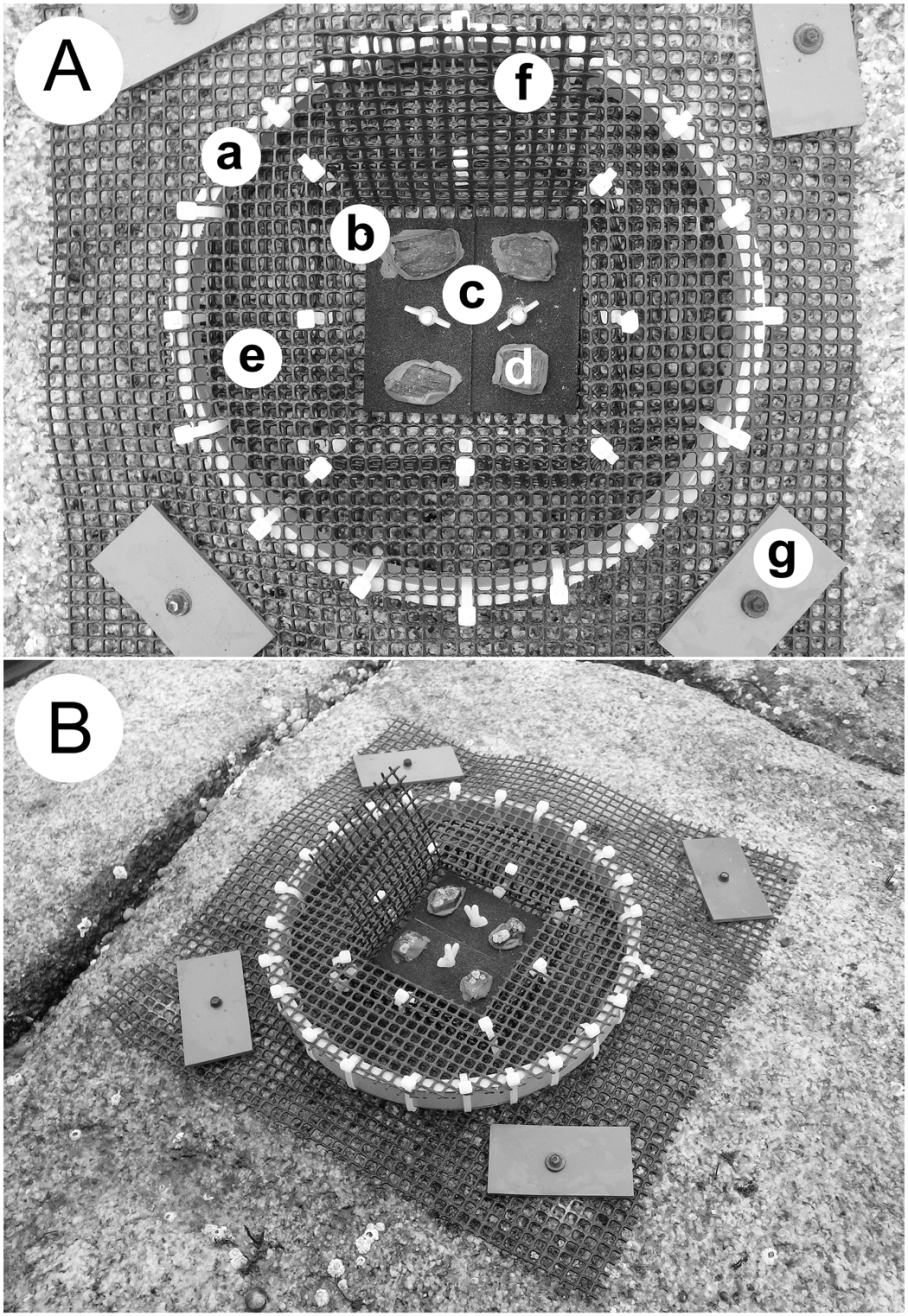
Experimental unit. (A) Top view of a cage, showing (a) the PVC ring of 25 cm in diameter, (b) the central compartment with (c) two barnacle recruitment tiles and (d) four small substrates (shown without adult barnacles), and (e) the peripheral compartment (shown without dogwhelks). The (f) top mesh of the central compartment is shown open to improve viewing of its internal components, but it remained closed with plastic cable ties during the experiment. The cage was secured with (g) screws and PVC plates to the substrate. (B) Side view of a cage (showing adult barnacles in the four substrates in the central compartment), exhibiting its limited height (2.5 cm) (Picture credits: Julius A. Ellrich).

The experimental unit (Fig 1) included a cage made of a PVC ring (25 cm in diameter and 2.5 cm tall) and plastic mesh (0.5 cm x 0.5 cm of opening size). Each cage was subdivided by mesh into a central compartment (12 cm x 12 cm) and a peripheral compartment (area = 347 cm^2^). We used the peripheral compartment to manipulate dogwhelk presence by either enclosing 10 dogwhelks (2.1–2.3 cm long) collected locally or by excluding dogwhelks. These values of dogwhelk density represent the natural density range on the studied coast (0–3 dogwhelks dm^-2^), which we determined using 60 random quadrats (40 cm x 40 cm). The central compartment included two contiguous PVC tiles (each one measuring 8.9 cm x 4.6 cm x 0.4 cm) covered with black tape with a sandpaper texture (Permastik self-adhesive anti-skid safety tread, RCR International, Boucherville, Quebec, Canada) to offer a suitable surface for barnacle recruitment. A pilot study indicated that such tiles are representative of natural rates of barnacle larval settlement, as the density of settled larvae during May did not differ between tiles (3 ± 1 individuals dm^-2^; mean ± SE, n = 12 tiles) and the natural rocky substrate (5 ± 2 individuals dm^-2^, n = 12 quadrats) (*t*_22_ = 0.809, *P* = 0.427). We also used the central compartment to manipulate adult barnacle presence. Each adult-present cage had four substrates (each one being 4-cm^2^ in area and 0.3–0.5 cm in height) hosting a total of 15 adult barnacles (0.5–1.5 cm in basal shell diameter and 0.3–0.5 cm in height) in the central compartment. These substrates were attached to the tiles with marine epoxy (A-788 Splash Zone Compound, Z-Spar, Los Angeles, CA, USA). An adult-absent cage had four such substrates without adult barnacles to eliminate the epoxy as a possible confounding factor. Because of the small size of the adult barnacles relative to the substrates and of the moderate wave exposure in the habitats, no major effects of adult barnacles on water motion were expected. We created these substrates by cutting out wood pieces with and without barnacles from a nearby dock. The density of adult barnacles in the central compartment (calculated based on the area of the two tiles) was representative of the studied shore. We secured the cages to the substrate with screws, washers, and plastic anchors placed into holes drilled into the substrate. We tightened the tiles to the bottom mesh of the cages with plastic screws, wing nuts, and washers. In our study habitats, dogwhelks were naturally feeding on a barnacle diet, as mussels (another possible prey for dogwhelks) [65] were largely absent.

Cues from adult barnacles [45,46] and dogwhelks [21] affect nearby cyprid settlement within centimeters. The caged dogwhelks could freely move inside the peripheral compartment and approach the recruitment tiles up to 1.5 cm. Thus, cyprids settling on the tiles were exposed to cues from adult barnacles and dogwhelks but not to physical contact with these predators. To exclude cyprid attraction by adult barnacles found outside the cages, we removed all adult barnacles from 40 cm x 40 cm areas around the center of each cage. We did not feed the caged dogwhelks during the experiment but, to prevent starvation, we replaced the dogwhelks every two weeks, releasing the removed individuals hundreds of meters away. We also removed any free-living dogwhelks found around the cages periodically. To exclude potential influences of seaweed mucus [19], canopy flow barriers [66] and canopy thermal and humidifying effects [49] on barnacle recruitment, we removed all seaweeds (mainly *Fucus vesiculosus* and some *Ascophyllum nodosum*) found around the cages. We started the experiment by setting up all treatments on the shore on 16 April 2012 and on 24 April 2013.

Barnacle recruits appeared for the first time on 30 April 2012 and on 9 May 2013. We measured barnacle recruit density on the tiles on 25 May 2012 and on 26 June 2013. On those dates, recruits had a basal diameter of 1–2 mm. No recruits appeared afterwards, so we sampled at the end of the 2012 and 2013 recruitment seasons, when maximum recruit densities were reached (S1 Dataset).

### Statistical Analysis

We conducted a nested, four-way analysis of variance (ANOVA) to test for the effects of dogwhelk cues (fixed factor with two levels: presence and absence), adult barnacles (fixed factor with two levels: presence and absence), year (random factor with two levels), and block (random factor with six levels, nested within year) on barnacle recruit density. We confirmed the homoscedasticity and normality assumptions using Cochran's *C*-test and Shapiro-Wilk test, respectively, after square-root transformation of the data. When nonsignificant results occurred for interactions involving random and fixed factors at *P* ≥ 0.25, we eliminated the corresponding sources of variation and pooled their sum of squares with the residual sum of squares to increase statistical power to test the remaining factors [67]. After running the final ANOVA following this procedure, we compared treatments using Tukey's Honestly Significant Difference (HSD) tests. We conducted the analyses with SPSS 18.

### Ethics Statement

We did the experiment in public-access marine intertidal habitats. The species that we used for the study, dogwhelks (*Nucella lapillus*) and barnacles (*Semibalanus balanoides*), are very abundant and not endangered or protected. Thus, neither a permit nor ethics approval was required for our research.

## Results

**Fig 2:**
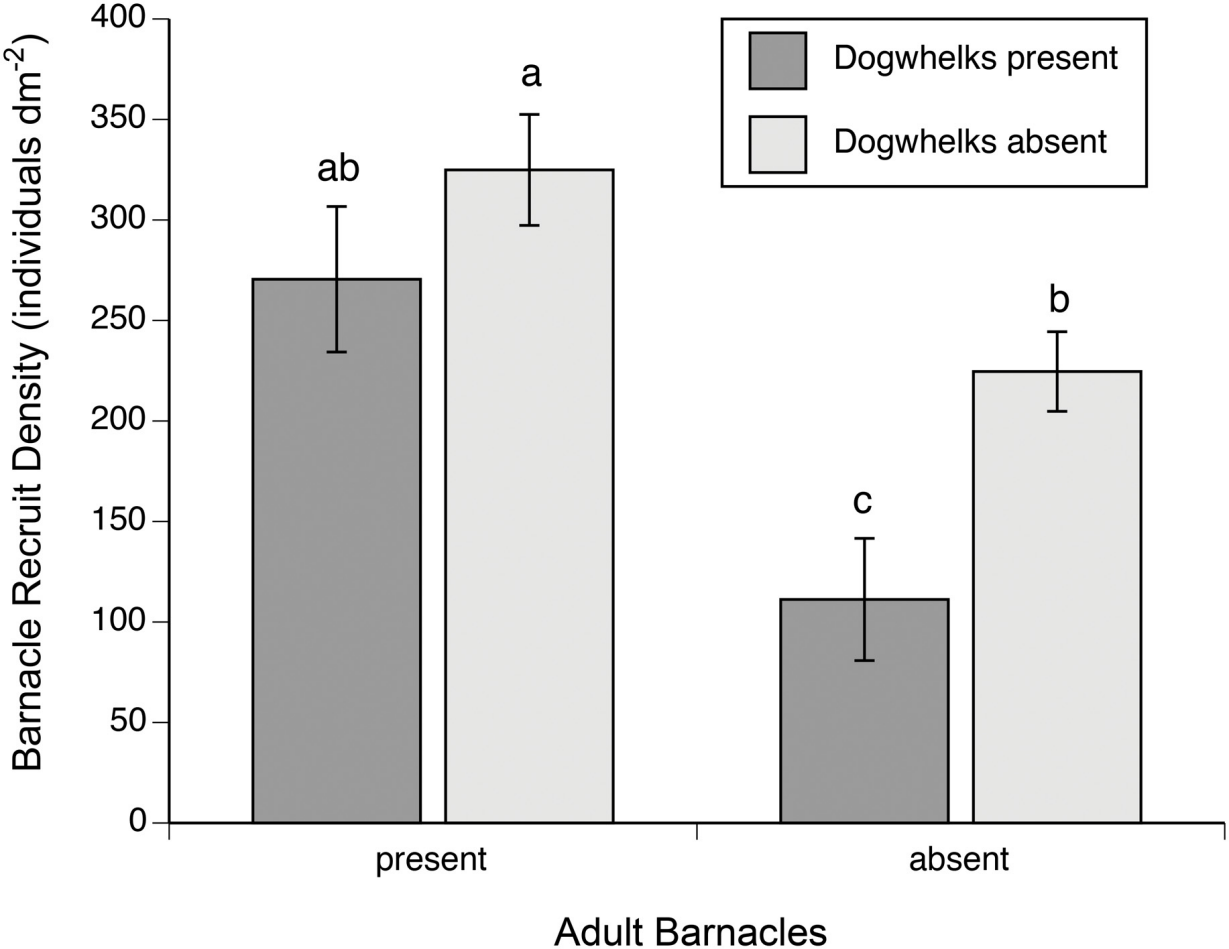
Barnacle recruit density (mean ± SE) in the presence and absence of nearby dogwhelks and adult barnacles. Significant differences between treatments (*P* < 0.05) are indicated when the two corresponding bars do not share the same letter.

The "year x dogwhelks", "year x adult barnacles", "dogwhelks x block(year)" and "dogwhelks x adult barnacles x block(year)" interactions exhibited *P* values higher than 0.25 in the first ANOVA (S1 Table). After pooling the sum of squares of those sources of variation with the residual sum of squares, the second ANOVA revealed that the "dogwhelk x adult barnacles x year" interaction then showed a *P* value higher than 0.25 (S2 Table). After a second step of sum-of-squares pooling, the final (third) ANOVA indicated that the presence of dogwhelks and adult barnacles significantly affected barnacle recruit density (Table 1). As the interaction between those two factors was also significant (Table 1), Tukey HSD tests compared the four corresponding treatments. Regardless of the nearby presence or absence of dogwhelks, adult barnacles significantly enhanced barnacle recruit density, by 44% on average combining both dogwhelk treatments (Fig 2). In turn, adult barnacle presence affected the expression of dogwhelk NCEs on barnacle recruitment. In the absence of adult barnacles, the nearby presence of dogwhelks significantly limited (Tukey HSD test, *P* < 0.001) barnacle recruit density (by 51% on average), but the presence of adult barnacles prevented dogwhelks from having any NCEs on barnacle recruit density (Tukey HSD test, *P* = 0.571; Fig 2), supporting this study’s hypothesis. The factor "year" and the "adult barnacles x block(year)" interaction were not significant (Table 1). The factor "block(year)" was significant, but this result merely tells that barnacle recruit density differed among blocks, the important result being that blocks did not interact with other factors, indicating that the interactive effects of dogwhelks and adult barnacles summarized above were spatially consistent on the shore.

**Table 1:** Summary results of the final ANOVA on barnacle recruit density.

Source of variation	df	MS	*F*	*P*
Dogwhelks	1	210.102	14.954	**<0.001**
Adult barnacles	1	536.024	20.560	**0.001**
Dogwhelks x Adult barnacles	1	59.218	4.215	**0.044**
Year	1	0.170	0.002	0.964
Block(Year)	10	78.309	3.001	**0.043**
Adult barnacles x Block(Year)	11	26.093	1.857	0.061
Pooled	69	14.050		

Results of the final (third) ANOVA that tested the effects of dogwhelk presence ("Dogwhelks"), adult barnacle presence ("Adult barnacles"), year ("Year"), and blocks nested within year ("Block(Year)") on barnacle recruit density on the Atlantic coast of Nova Scotia, Canada, at the end of the 2012 and 2013 recruitment seasons. The term "Pooled" refers to the residual source of variation in the first ANOVA plus the variation for the sources that were nonsignificant with *P* ≥ 0.25 in the first and second ANOVA, which are summarized in the S1 and S2 Tables. Significant *P* values (*P* < 0.05) are highlighted in boldface.

## Discussion

This study has revealed that the presence of adult barnacles prevents the nonconsumptive limitation that dogwhelks would otherwise exert on barnacle recruitment. This is an important finding because, as predator NCEs may influence prey populations more than consumptive effects [12–17], it is relevant to unravel the factors that influence the occurrence of NCEs [18]. Our results may be explained by considering the known role of dogwhelk and adult barnacle cues. On the one hand, in the absence of adult barnacles, dogwhelk cues have been found to limit barnacle larval settlement [19,21] and subsequent recruitment [22], as such cues are an indication of predation risk in benthic habitats. However, adult barnacles attract conspecific larvae that are seeking settlement [37–40] through chemical [41–45] and visual cues [46]. Correspondingly, our experiment has shown that adult barnacle presence increases barnacle recruitment, which was the case regardless of the presence or absence of dogwhelks. Therefore, the lack of dogwhelk NCEs on barnacle recruitment in the presence of adult barnacles likely resulted from the influence of the attractive cues from the adult barnacles. In the presence of barnacle adults, cyprid larvae possibly did sense dogwhelk cues when these predators were present, but the adult barnacle cues seemingly had a more prominent role in the settlement behavior of barnacle larvae. Adult barnacle cues may have indicated to larvae that abiotic and biotic conditions were suitable for post-settlement growth and reproduction [33].

Future research could investigate if the relative density of dogwhelks and adult barnacles may influence the occurrence of dogwhelk NCEs on barnacle recruitment. For example, a higher dogwhelk density than used in our experiment might trigger NCEs on barnacle recruitment under the adult barnacle density we used. This could be so because studies with other species have shown that predator NCEs on prey behavior may intensify with predator density through the increase of predator cues in the environment [68–70]. Dogwhelk density has already been found to influence the occurrence of NCEs on barnacle recruitment in the absence of adult barnacles [71]. On the other hand, a lower adult barnacle density than used in our experiment might limit, but not neutralize, dogwhelk NCEs on barnacle recruitment for the dogwhelk density we used. This could be the case because microcosm experiments with other species have found that, for a given predator density, the intensity of predator NCEs on prey activity and growth is negatively related to prey density [72,73]. Because of the convenient body size of dogwhelks and barnacles for field experimentation, this model predator—prey system could help to further advance the theory about density influences on predator NCEs on prey demography.

Besides barnacles, many invertebrate species show attraction of conspecific larvae by adult organisms, including other arthropod species and species of molluscs, polychaetes, echinoderms, and tunicates [26–31]. Thus, reduced or absent predator NCEs on the recruitment of those species in the presence of adult conspecifics could be a common phenomenon. Exceptions could be cannibalistic species in which adults or juveniles consume conspecific larvae. For instance, in cannibalistic crabs, conspecific presence may have neutral [20] or negative [3] NCEs on larval settlement, suggesting that conspecific presence would not neutralize negative NCEs from heterospecific predators on prey recruitment. A thorough understanding of the interactive effects of predator and adult prey density on prey recruitment could be gained through field experiments using prey species spanning a range of adult influences on larval settlement behavior.

## Supporting Information

S1 Dataset Barnacle recruit density (individuals dm^-2^) in the presence and absence of dogwhelks and adult barnacles. (XLSX)

S1 Table Results of the first ANOVA that preceded the second ANOVA that is summarized in the S2 Table. The first ANOVA tested the effects of the nearby presence of dogwhelks (denoted as "D"), presence of adult barnacles ("A"), year ("Y"), and block nested within year (“B(Y)”) on barnacle recruit density on the Atlantic coast of Nova Scotia, Canada, at the end of the 2012 and 2013 barnacle recruitment seasons.
(DOCX)

S2 Table Results of the second ANOVA that preceded the final ANOVA that is summarized in Table 1. The second ANOVA tested the effects of the nearby presence of dogwhelks (denoted as "D"), presence of adult barnacles ("A"), year ("Y"), and block nested within year (“Block(Year)”) on barnacle recruit density on the Atlantic coast of Nova Scotia, Canada, at the end of the 2012 and 2013 barnacle recruitment seasons. The term "Pooled" refers to the residual source of variation in the first ANOVA plus the variation for the sources that were nonsignificant with *P* ≥ 0.25 in the first ANOVA.
(DOCX)
