# Supplementary material for: Adult Prey Neutralizes Predator Nonconsumptive Limitation of Prey Recruitment
Source: PLoS One. 2016 Apr 28;11(4):e0154572. doi: 10.1371/journal.pone.0154572 (PMC4849580; doi:10.1371/journal.pone.0154572)
Supplement: S1 Table — The first ANOVA tested the effects of the nearby presence of dogwhelks (denoted as "D"), presence of adult barnacles ("A"), year ("Y"), and block nested within year (“B(Y)”) on barnacle recruit density on the Atlantic coast of Nova Scotia, Canada, at the end of the 2012 and 2013 barnacle recruitment seasons. (DOCX) [file pone.0154572.s002.docx]

**S1 Table** **Results of the first ANOVA that preceded the second ANOVA that is summarized in the S2 Table.**

| **Source of variation** | **df** | **MS** | ***F*** | ***P*** |
| --- | --- | --- | --- | --- |
| D | 1 | 209.435 | 83.566 | 0.069 |
| A | 1 | 531.501 | 14.359 | 0.164 |
| D x A | 1 | 59.199 | 2.674 | 0.349 |
| Y | 1 | 0.188 | 0.003 | 0.964 |
| Y x D | 1 | 2.507 | 0.108 | 0.796 |
| Y x A | 1 | 37.015 | 1.023 | 0.404 |
| Y x D x A | 1 | 22.141 | 2.173 | 0.171 |
| B(Y) | 10 | 78.165 | 3.113 | 0.063 |
| D x B(Y) | 10 | 11.266 | 1.107 | 0.437 |
| A x B(Y) | 10 | 24.016 | 2.361 | 0.096 |
| D x A x B(Y) | 10 | 10.173 | 0.655 | 0.759 |
| Residual | 47 | 15.519 |  |  |

The first ANOVA tested the effects of the nearby presence of dogwhelks (denoted as "D"), presence of adult barnacles ("A"), year ("Y"), and block nested within year (“B(Y)”) on barnacle recruit density on the Atlantic coast of Nova Scotia, Canada, at the end of the 2012 and 2013 barnacle recruitment seasons.
