# Supplementary material for: Adult Prey Neutralizes Predator Nonconsumptive Limitation of Prey Recruitment
Source: PLoS One. 2016 Apr 28;11(4):e0154572. doi: 10.1371/journal.pone.0154572 (PMC4849580; doi:10.1371/journal.pone.0154572)
Supplement: S2 Table — The second ANOVA tested the effects of the nearby presence of dogwhelks (denoted as "D"), presence of adult barnacles ("A"), year ("Y"), and block nested within year (“Block(Year)”) on barnacle recruit density on the Atlantic coast of Nova Scotia, Canada, at the end of the 2012 and 2013 barnacle recruitment seasons. The term "Pooled" refers to the residual source of variation in the first ANOVA plus the variation for the sources that were nonsignificant with P ≥ 0.25 in the first ANOVA. (DOCX) [file pone.0154572.s003.docx]

**S2 Table** **Results of the second ANOVA that preceded the final ANOVA that is summarized in Table 1.**

| **Source of variation** | **df** | **MS** | ***F*** | ***P*** |
| --- | --- | --- | --- | --- |
| D | 1 | 208.663 | 16.390 | 0.056 |
| A | 1 | 537.968 | 23.667 | **0.010** |
| D x A | 1 | 58.476 | 4.593 | 0.165 |
| Y | 1 | 0.133 | 0.002 | 0.968 |
| Y x A x D | 2 | 12.731 | 0.904 | 0.410 |
| B(Y) | 10 | 78.418 | 3.253 | **0.038** |
| A x B(Y) | 10 | 24.106 | 1.711 | 0.096 |
| Pooled | 67 | 14.089 |  |  |

The second ANOVA tested the effects of the nearby presence of dogwhelks (denoted as "D"), presence of adult barnacles ("A"), year ("Y"), and block nested within year (“Block(Year)”) on barnacle recruit density on the Atlantic coast of Nova Scotia, Canada, at the end of the 2012 and 2013 barnacle recruitment seasons. The term "Pooled" refers to the residual source of variation in the first ANOVA plus the variation for the sources that were nonsignificant with *P* ≥ 0.25 in the first ANOVA.
